# Clinical Profile and Outcome of Severe Acute Respiratory Illness (SARI) in Children Amidst the COVID-19 Pandemic

**DOI:** 10.7759/cureus.61902

**Published:** 2024-06-07

**Authors:** Reena Jain, Shivanjali Sood, Shivani Randev, Pankaj Kumar, Vishal Guglani

**Affiliations:** 1 Pediatrics, Government Medical College and Hospital, Chandigarh, Chandigarh, IND

**Keywords:** sari, coronavirus, sars-cov-2, covid, severe acute respiratory infection

## Abstract

Background and objectives: Beginning in December 2019, COVID-19 rapidly emerged as a global pandemic. Though its severity in children was reported to be less than that in adults, data on its epidemiology in relation to severe acute respiratory illness (SARI) caused by other microbes needed to be generated. This study compares the clinical profile and outcome of children hospitalized with COVID-19-positive and negative SARI.

Methods: This is a prospective observational analytical study involving children 1 month to 18 years old, hospitalized with COVID-19-positive and negative SARI during the pandemic. All eligible patients were enrolled after obtaining informed parental consent. Their clinical manifestations, investigations, and outcomes were documented on a predesigned case record form. A nasopharyngeal swab sample for COVID-19 reverse transcription polymerase chain reaction was sent, and results were noted.

Results: From May 2020 to July 2021, 267 children were hospitalized with a diagnosis of SARI. Out of these, 146 (54.7%) were boys and 78.7% were under five years of age. Other presentations included fever and cough, breathlessness, nausea, vomiting, diarrhea, rash, seizures, and altered sensorium. Twenty-eight patients (10.5%) tested positive for COVID-19. COVID-19 patients were similar in terms of demographic characteristics and presenting symptoms to non-COVID-19 patients but had a lower absolute lymphocyte count (p = 0.019) and higher serum alanine transaminase levels (p = 0.013). Acute respiratory distress syndrome (OR, 4.3; 95% CI, 1.8-10.0), shock (OR, 3.9; 95% CI, 1.9-7.9), and need for intensive care unit admission (OR, 9.9; 95% CI, 6.9-14) were more common in COVID-19 SARI patients. Death occurred in 18% of COVID-19 and 9% of non-COVID-19 patients (p = 0.07). SARI nonsurvivors had significantly lower blood pH and platelet counts than survivors.

Conclusions: Comparison of COVID-19-positive and negative SARI patients showed subtle differences between the two groups, with COVID-19-positive children having an increased severity of illness. Also, laboratory evidence of multiorgan dysfunction at admission was associated with higher mortality.

## Introduction

Acute respiratory infections (ARIs) are a leading cause of morbidity and mortality in children and pose a significant public health problem. The precise magnitude of childhood ARIs in India is unknown, and most available data are based on small-scale community and hospital-based studies. A recent systematic review estimated the disease burden to be between 0.03 and 0.52 episodes per child per year; nearly 10% of these episodes are due to pneumonia [1]. Viruses cause most cases of respiratory infections in children. The potential viral pathogens of ARIs include seasonal A and B influenza viruses, human metapneumovirus, human rhinovirus, human adenovirus, human parainfluenza viruses, respiratory syncytial virus, human bocavirus, human coronaviruses (CoVs), and enterovirus [2,3]. Influenza and CoVs can undergo mutations, leading to the emergence of novel strains, which result in severe outbreaks. The current COVID-19 pandemic has resulted from a novel mutated CoV called "severe acute respiratory syndrome coronavirus-2" (SARS-CoV-2, COVID-19), which originated in Wuhan, China, in 2019.

Children with COVID-19 infection have shown a wide range of symptomatology, from asymptomatic infection to severe illness requiring intensive care unit (ICU) admission. The common symptoms are like any ARI, such as fever, cough, sore throat, and rhinorrhea with or without congestion or headache. A small proportion of patients present with respiratory distress and require hospitalization, and few develop severe lower respiratory tract infections leading to complications like acute respiratory distress syndrome (ARDS), shock, and multiorgan dysfunction syndrome (MODS) [4]. We undertook this prospective observational study to evaluate the clinical profile and outcomes of pediatric patients hospitalized with the diagnosis of severe ARI during the pandemic and to carry out a comparative analysis of clinical characteristics between COVID-19 and non-COVID-19 severe acute respiratory infection (SARI) patients.

## Materials and methods

This prospective study was conducted at a tertiary-care pediatric teaching hospital in Northern India. During the SARS-CoV-2 outbreak, a dedicated area was made operational for SARI patients, with segregated wards for COVID-19-positive and negative SARI patients. This area was well equipped with intensive care equipment. All patients between 1 month and 18 years of age who presented with a primary diagnosis of SARI as per WHO definition (an ARI with a history of fever or documented temperature of ≥38°C or higher, cough onset within the last 10 days, and necessitating hospitalization) were admitted in the SARI ward. A nasopharyngeal swab sample for COVID-19 reverse transcription polymerase chain reaction (RT-PCR) was collected from all patients upon admission. All patients received emergency treatment in the common area of the SARI ward and were further managed in their respective areas (COVID-19 or non-COVID-19) based on the RT-PCR report. All patients were subjected to investigations like complete blood count, blood culture, liver and renal function tests, blood gas analysis, and chest radiography. COVID-19 patients were treated according to the WHO guidelines, and all non-COVID-19 patients were managed according to their respective diagnoses.

Patients admitted to the SARI ward from May 2020 to July 2021 were enrolled in this study after obtaining informed consent. The Institutional Ethics Committee granted ethics approval. Demographic data, symptoms, clinical findings, investigations, treatment received (antimicrobials, bronchodilators, inotropes, oxygen, and need for ventilation), duration of hospital stay, and outcomes were documented on a prestructured pro forma. The data were then analyzed to examine all patients' demographic and clinical profiles and differentiate between the COVID-19 and non-COVID-19 groups.

Statistical analysis

Quantitative variables were analyzed using measures of central tendency (mean, median). Qualitative data were analyzed using frequencies and proportions. Means were compared using a t-test for parametric and the Mann-Whitney test for nonparametric data. Dichotomous variables were compared using the chi-square test. The statistical significance was defined with a p value (p < 0.05). Multivariate analysis was done using logistic regression. Statistical analysis was done using IBM SPSS Statistics version 26 (IBM Corporation, Armonk, NY, USA).

## Results

From May 2020 to July 2021, 267 children were hospitalized with a diagnosis of SARI. Out of these, 146 (54.7%) were boys. Most of the children were under five years old (78.7%), and almost half (48.7%) were infants (Table 1).

**Table 1:** Baseline characteristics of enrolled patients IQR: interquartile range; SD: standard deviation; BMI: body mass index

Characteristics	Number (N = 267), n (%)
Age in months, median (IQR), mean ± SD	12 (4-48), 40.58 ± 56.85
Age group (years)	
<1	130 (48.7%)
1-5	80 (30%)
5-10	24 (9.0%)
>10	33 (12.3%)
Gender: boys	146 (54.68)
Anthropometry: z-score (mean ± SD)	
Weight for age	-0.59 ± 0.74
Height/length for age	-0.53 ± 0.67
Weight for height (up to five years)	0.52 ± 0.92
BMI (more than five years)	0.78 ± 0.79

Out of the 267 children, 28 (10.5%) tested positive for the COVID-19 virus and were moved to the COVID-19 ward for treatment, while those who tested negative were managed in a non-COVID-19 SARI ward. Among non-COVID-19 patients, the most common diagnosis was pneumonia (20%), followed by wheeze-associated lower respiratory infection (12%), sepsis (12.5%), acute bronchiolitis (11%), and acute exacerbation of asthma (7.5%). Almost 18% of non-COVID-19 SARI patients had an underlying comorbid condition, most commonly asthma, tuberculosis, and diabetes. Out of these, 215 patients (90%) showed improvement and were discharged, while 21 patients passed away.

Among children with COVID-19, the mean duration of fever was 1.93 ± 1.82 days. The most common symptoms after fever and cough were shortness of breath (68%), diarrhea (25%), nausea/vomiting (18%), and altered sensorium/seizures (7%). Underlying comorbid conditions were reported in around 21% of patients, which included kidney disease (nephrotic syndrome, C3 glomerulopathy), storage disorder (Niemann-Pick disease), and neurological disorder (tuberous sclerosis and epilepsy) (Table 2).

**Table 2:** Presenting clinical and baseline laboratory parameters of COVID-19 and non-COVID-19 SARI patients a: Median (IQR) b: Mean (standard deviation). c: n (%) SpO2: oxygen saturation; DM: diabetes mellitus; WBC: white blood cells; ANC: absolute neutrophil count; ALC: absolute lymphocyte count; ALT: alanine aminotransferase; AST: aspartate aminotransferase; ULN: upper limit of normal; SARI: severe acute respiratory illness

Characteristics	Non-COVID-19 (N = 239), n (%)	COVID-19 (N = 28), n (%)
Age^a^ (months)	12 (3.5-48)	11 (3-40)
Gender: boys	132 (55.2%)	14 (50%)
Clinical characteristics		
Fever	239 (100)	28 (100)
Duration of fever (days)^b^	2.06 ± 2.20	1.93 ± 1.82
Shortness of breath	181 (75.7)	19 (68)
Nausea and vomiting	12 (5.0)	5 (18)
Diarrhea	81 (33.9)	7 (25)
Rash	45 (18.8)	0 (0)
Seizure/altered sensorium	13 (5.4)	2 (7.1)
Tachypnea	239 (100)	28 (100)
Chest auscultation (adventitious sounds)		
Wheeze	28 (11.7)	7 (25.0)
Crepitations	31 (13)	1 (3.6)
Hypoxemia (SpO_2_ < 94%)	158 (66)	16 (57.1)
Organomegaly (hepatomegaly/splenomegaly)	10 (4.1)	4 (14.2)
Comorbidities	43 (18.0)	6 (21.4)
Asthma	18 (7.5)	0
Nephrotic syndrome	6 (2.5)	1 (3.6)
Type 1 DM	5 (2.0)	0
Neurological disorder	5 (2.0)	2 (7.1)
Tuberculosis	6 (2.5)	0
Storage disorder	0	2 (7.1)
Others	3 (1.3)	1 (3.6)
Laboratory parameters		
WBC count (×10^9^/L)^a^	12 (4-16)	118 (76-137)
Leukocytosis (>11 × 10^9^/L)^c^	134 (56%)	17 (60.7%)
Leucopenia (<4 × 10^9^/L)^c^	6 (2.5%)	4 (14.2%)
Differential leucocyte count		
Neutrophil (%)^a^	67 (57-78)	68 (59-75)
ANC/mm^3^ ^a^	7,700 (5,656-11,200)	7,248 (4,308-9,691)
ANC < 1,500^b^	1 (0.004)	3 (10.7%)
ALC/mm^3 a^	3,441 (2,500-4,550)	2,799 (1,920-3,673)
ALC < 1,500^b^	9 (3.7%)	4 (14.2%)
Platelet count (×10^9^/L)^a^	245 (180-343)	244 (154-339)
Thrombocytopenia (<150 × 10^9^/L)^b^	41 (17.3)	6 (21.4)
Serum ALT (IU/L)^a^	25 (16-38)	33 (24-45.7)
Serum AST (IU/L)^a^	41 (33-49.5)	45.5 (38.5-76.5)
Transaminitis (ALT > 2ULN)^b^	16/239 (6.7%)	4/28 (14.2%)
Serum creatinine (mg/dL)^b^	0.6 (0.5-0.8)	0.7 (0.6-0.8)
Abnormal chest radiograph^b^	78 (32.6%)	19 (67%)

Hypoxemia was observed in 57% of children at the time of admission, and 92% of patients needed respiratory support: only supplemental oxygen (32%), noninvasive ventilation/continuous positive airway pressure (42%), and invasive ventilation (18%). Radiologically confirmed pneumonia was found in almost 40% of patients, and 14% developed ARDS. The most frequent complication observed in COVID-19 patients was shock requiring vasoactive support (28.6%), followed by acute kidney injury (18%), elevated transaminases (14%), ARDS (14%), encephalopathy (7%), coagulopathy (7%), and multisystem inflammatory syndrome in children (3.6%) (Table 3). Three-fourths of the children had abnormal blood counts: leukocytosis (60%), leucopenia (14%), lymphopenia (14%), and neutropenia (10.7%) (Table 3).

**Table 3:** Clinical course and outcomes of COVID-19 and non-COVID-19 SARI patients *Mean (SD) CPAP: continuous positive airway pressure; NIV: noninvasive ventilation; ARDS: acute respiratory distress syndrome; MIS-C: multisystem inflammatory syndrome in children; LAMA: left against medical advice; SARI: severe acute respiratory illness; SD: standard deviation

Characteristics	Non-COVID-19 (N = 239), n (%)	COVID-19 (N = 28), n (%)
Duration of hospital stay in days*	7 (5.32)	9 (5.01)
Treatment received		
Antibiotics	167 (69.9)	23 (82.1)
Bronchodilators	97 (40.6)	14 (50)
Systemic steroids	73 (30.5)	13 (46.4)
Respiratory support		
Nasal CPAP	89 (37.2)	11 (39.2)
NIV	10 (4.2)	1 (3.0)
Invasive ventilation	28 (11.7)	5 (17.8)
Complications		
ARDS	6 (2.5)	4 (14.2)
Encephalopathy/seizures	13 (5.4)	2 (7.1)
Elevated transaminases	16 (6.7)	4 (14.2)
Acute kidney injury	30 (12.6)	5 (17.9)
MIS-C	4 (1.6)	1 (3.6)
Shock requiring vasoactive support	17 (7.1)	8 (28.6)
Coagulopathy	9 (3.8)	2 (7.1)
Outcome		
Discharge	215 (90)	22 (78.6)
LAMA	4 (1.5)	0
Death	21 (8.8)	5 (17.9)

A comparison of clinical and laboratory parameters between COVID-19-positive and negative patients showed no significant differences in age, gender distribution, and symptomatology (Table 2). On investigations, COVID-19 SARI patients had a lower absolute lymphocyte count (p = 0.019) and higher serum alanine aminotransferase (ALT) levels (p = 0.013) compared to the non-COVID-19 group. In terms of severity of illness, ARDS was more common in COVID-19-positive SARI patients (OR, 4.3; 95% CI, 1.8-10.0), as was shock requiring inotropic support (OR, 3.9; 95% CI, 1.9-7.9) and need for admission to ICU (OR, 9.9; 95% CI, 6.9-14). The illness resulted in death in 18% of COVID-19-positive patients compared to approximately 9% of COVID-19-negative SARI patients, but the difference was not statistically significant (p = 0.07; OR, 2; 95% CI, 0.9-4.9). Among the five COVID-19 patients (18%) who passed away, three were boys. Four patients were under two years of age, and one was 18 years old. Four had COVID-19 pneumonia with ARDS, and one patient had underlying chronic kidney disease.

A comparative analysis was also done between the survivors of SARI and those who succumbed to their illness during their hospital stay (Table 4). It was noted that the nonsurvivors had significantly higher total leukocyte and absolute neutrophil counts and lower platelet counts than the survivors in the baseline hemogram. The nonsurvivors also had significantly higher serum transaminases, urea, and creatinine values and lower blood pH in samples drawn at hospital admission. However, on multivariate analysis, only lower platelet counts (p = 0.012) and lower pH (p = 0.0) had a significant association with mortality.

**Table 4:** Univariate analysis of clinical and laboratory parameters at admission in survivors and nonsurvivors IQR: interquartile range; AST: aspartate transaminase; ALT: alanine transaminase; ABG: arterial blood gas; PaO2: arterial oxygen pressure; PCO2: partial pressure of carbon dioxide

Characteristic [median (IQR)]	Survivors (N = 237)	Nonsurvivors (N = 26)	P value
Age (months)	12 (4-48)	11.5 (3-24)	0.9
Weight (kg)	10 (5-16.2)	9.6 (5.5-15)	0.9
Gender, n (%)			
Boys (144)	131 (90.97)	13 (9.0)	0.4
Girls (119)	106 (89.07)	13 (10.9)	
Fever duration before admission (days)	1 (1-2)	1 (1-4)	0.4
SpO_2 _at admission (%)	94 (90-98)	91 (89-96)	0.1
Hemoglobin (g/L)	102 (94-110)	98 (95-104)	0.09
Total leucocyte count (×10^9^/L)	11.9 (8.5-15.2)	14.15 (10.2-22.7)	0.01
Absolute neutrophil count (×10^9^/L)	7.56 (5.37-10.30)	11.15 (6.50-15.60)	0.004
Platelet count (×10^9^/L)	245 (168-342)	172 (88-234)	0.03
AST (U/L)	39 (32-50)	45 (39-102)	0.04
ALT (U/L)	24 (16-38)	35 (26-68)	0.001
Urea (mg/dL)	22 (16-35)	30 (20-76)	0.04
Creatinine (mg/dL)	0.6 (0.5-0.8)	0.8 (0.7-1)	0.13
ABG analysis			
pH	7.35 (7.31-7.42)	7.33 (7.11-7.41)	0.05
PaO_2 _(mm Hg)	77.8 (56.2-96.7)	68 (48.4-85)	0.07
PCO_2 _(mm Hg)	34.7 (30.7-39.7)	39.65 (30-48.4)	0.07
HCO_3 _(mEq/L)	19.3 (14.5-22.1)	16.95 (11.9-22.7)	0.08
Lactate (mmol/L)	1.5 (1-2.2)	1.8 (1.2-3.7)	0.13

## Discussion

This study, conducted during the COVID-19 pandemic, compared the manifestations and outcomes of children hospitalized with COVID-19-positive SARI to those who tested negative for COVID-19. COVID-19 was responsible for 10.5% of children hospitalized with a diagnosis of SARI in our region. Almost half of the children with SARI were infants, and approximately 80% were under five years of age. A study on ARIs in children before the COVID-19 pandemic concluded that 6.5% of ARIs in children were due to CoVs, and no differences between demographic characters and clinical presentation were noted between CoV and other virus ARIs [2,5]. In another study on children hospitalized with acute lower respiratory infection (ALRI), CoVs were detected in respiratory samples of 4.6% of children and accounted for 9.5% of viral ALRIs [6]. The onset of the pandemic changed the epidemiology, and the relative proportion of ARIs in children due to CoVs increased. What remained unchanged was the fact that hospitalization with SARI had always been more common in younger children [7]. A greater proinflammatory cytokine response in younger children has been considered to cause greater severity of infection in them [8].

In the current study, COVID-19-positive children were similar to those who tested negative in terms of age and gender distribution, as well as in terms of clinical presentation. However, they were more likely to have laboratory abnormalities like lymphopenia, elevated serum ALT levels, and abnormal chest radiographs. They also had a higher risk of developing complications like ARDS and shock, and a higher proportion of them required intensive care. Overall, approximately 10% of children hospitalized due to SARI succumbed to the illness. Although the mortality rate in COVID-19 patients was 18%, almost double that in non-COVID-19 patients, the difference did not reach statistical significance. COVID-19 in children has been considered to be a milder illness compared to that in adults. However, the current study findings suggest that COVID-19 SARI has a greater severity of illness than SARI due to other viral infections, even in children. Several other studies also report similar findings despite wide variations in severity indices.

In a study by Singh et al. during the COVID-19 pandemic, SARS-CoV-2 positivity in pediatric SARI patients was seen in 28.3% of patients, much higher than the proportion of 10.5% seen by us [9]. The difference could have been due to differences in the number of COVID-19 cases at diverse time points in different geographical areas due to variations in containment strategies. Almost half the COVID-19 SARI patients seen by them were under five years of age, radiological pneumonia was seen in 44% of children, and ARDS was the most frequently seen complication.

Another study from our region compared clinical characteristics in hospitalized children who tested positive for SARS-CoV-2 during two different waves of the pandemic in the country. They observed almost 39% of the children to be infants, 30% of children required intensive care, 21% needed invasive ventilation, and 13.5% of children died. However, the proportion of children with comorbidities in their cohort was 66%, much higher than ours, and almost 70% of children were determined to be coincidentally COVID-19-positive [10].

In a registry of hospitalized COVID-19 cases from Germany during a similar period, infants constituted 42% of all pediatric patients. Comorbidities were seen in at least 27% of patients [11]. ARDS occurred in 0.8% of children, and mortality happened in 0.5%, less than half of which was directly attributed to SARS-CoV-2. The remarkable differences in their observations from ours could be because theirs was a multicentric study with variable criteria for hospitalization. Only 15% of patients in their cohort had manifestations of lower respiratory infection, and almost 11% had asymptomatic COVID-19 infection.

The various studies on the clinical profile of children hospitalized with COVID-19 infection have reported variable indices of illness severity; the need for intensive care varied from 17% to 25% among hospitalized children, with the requirement for mechanical ventilation ranging from 4% to 20%, and mortality ranging from 3% to 20% [12-16]. These rates, however, need to be interpreted with caution as most studies differ in several aspects, such as indications for hospitalization, variable government policies regarding hospitalization and discharge in COVID-19 patients, time period of the study as different waves of the pandemic could have differences in illness severity as well as availability of health infrastructure to manage the cases, and level of the center reporting the data as proportion of patients with comorbidities is higher in referral hospitals and severity of illness might be due to the nature of comorbidities. A similar study in adult patients showed significantly higher mortality in COVID-19-positive SARI patients compared to non-COVID-19 SARI patients [17].

The current study noted significant differences in baseline laboratory values for leucocyte count, platelet count, hepatic transaminases, urea, creatinine, and blood pH between children who survived and those who succumbed to their illness. Since all these point toward the involvement of multiple body systems, it probably implies that multiorgan dysfunction had already set in by the time these children were hospitalized. Similar results were seen in another study from our region on non-COVID-19 SARI patients, where it was noted that elevated liver enzymes, hypoxemia at admission, and MODS predicted death [18]. This could mean that evidence of MODS on baseline evaluation at the time of hospital admission may be used to triage patients in case of limited resources, giving them a better chance at survival.

The main strengths of this study are that it is a prospective study that directly compares SARS-CoV-2-related SARI with that due to other infections at similar time points and in a situation with similar resources for affected patients. The major limitation is that it is a single-center study representing only a part of the country. Also, testing respiratory specimens for other common respiratory viruses could have added more value as some patients might be coinfected with multiple viruses.

## Conclusions

The current study concluded that COVID-19-positive and negative SARI patients have subtle differences in clinical presentation, with COVID-19-positive children having an increased severity of illness and a greater need for intensive care. We also found that hospitalized SARI patients who had laboratory evidence of multiorgan dysfunction at the time of hospital admission have a higher risk of mortality. Hence, any evidence of multiorgan involvement in these patients in baseline workup may justify more intensive treatment.

Viruses like COVID-19 can mutate over time, leading to new variants with subtle to drastic differences in clinical manifestations and, hence, variable public health implications. Continuing comparative research enhances our understanding of the unique features of the disease; the knowledge guiding the development of evidence-based strategies for disease surveillance, outbreak detection, and informed clinical decision-making.
